# Killer instincts: natural killer cells as multifactorial cancer immunotherapy

**DOI:** 10.3389/fimmu.2023.1269614

**Published:** 2023-11-28

**Authors:** Sarah Nersesian, Emily B. Carter, Stacey N. Lee, Lauren P. Westhaver, Jeanette E. Boudreau

**Affiliations:** ^1^ Department of Microbiology and Immunology, Dalhousie University, Halifax, NS, Canada; ^2^ Beatrice Hunter Cancer Research Institute, Halifax, NS, Canada; ^3^ Department of Pathology, Dalhousie University, Halifax, NS, Canada

**Keywords:** natural killer cells, killer immunoglobulin-like receptors (KIR), signal integration, cancer immunotherapy, innate lymphoid cells

## Abstract

Natural killer (NK) cells integrate heterogeneous signals for activation and inhibition using germline-encoded receptors. These receptors are stochastically co-expressed, and their concurrent engagement and signaling can adjust the sensitivity of individual cells to putative targets. Against cancers, which mutate and evolve under therapeutic and immunologic pressure, the diversity for recognition provided by NK cells may be key to comprehensive cancer control. NK cells are already being trialled as adoptive cell therapy and targets for immunotherapeutic agents. However, strategies to leverage their naturally occurring diversity and agility have not yet been developed. In this review, we discuss the receptors and signaling pathways through which signals for activation or inhibition are generated in NK cells, focusing on their roles in cancer and potential as targets for immunotherapies. Finally, we consider the impacts of receptor co-expression and the potential to engage multiple pathways of NK cell reactivity to maximize the scope and strength of antitumor activities.

## 1 Natural killer cell biology

Natural killer (NK) cells are agile lymphocytes capable of immune polarization and rapid responsiveness to eliminate virally infected or malignant cells. Though they were initially described for their ability to discern “self” from “non-self” cells based on expression of class I major histocompatibility molecules (MHC) (1), NK cells’ function and underlying molecular toolkit is now understood to be much broader. Upon receiving signals from healthy, stressed, infected, or transformed cells, and in response to environmental signals, NK cells either perform cytotoxicity, stimulate subsequent immunity, regulate immunity, or do nothing at all (2, 3). This “polyvalency” to integrate signals for activation and inhibition creates a unique form of diversity in the immune system by using germline-encoded receptors, making NK cells attractive candidates for cell-based cancer immunotherapies (4, 5).

NK cells differ from other group one innate lymphoid cells (ILCs) by their relatively high expression of the transcription factor eomesodermin (EOMES), and the ability to mediate direct cytolysis of target cells via degranulation to release perforin and granzyme (6). In contrast to other lymphocytes, NK cells do not express antigen-specific clonotypic receptors (1, 7–10). Instead, NK cells use a constellation of germline-encoded activating and inhibitory receptors (**Table 1**). These receptors are differentially expressed, co-expressed, and armed among the NK cells that comprise an individual’s NK cell repertoire, and can range from relatively immature and inexperienced to adaptive and highly functional cells. This creates diversity to respond to the extensively variable phenotypes created by transformation, infection, and disease (7, 10, 13). Indeed, though HLA loss eliminates a dominant inhibitory signal to NK cells, other inhibitory mechanisms within the tumor microenvironment can also interfere with NK cell inhibition; these include metabolic dysregulation (14), inhibitory cytokine release (15), regulatory immune populations (16), and even biophysical properties of the cancer cells themselves (17).

**Table 1:** NK cell receptors, cognate ligands and their known signaling domains/ proximal adapters.

Receptor	*Gene*	Ligand	Signaling domains & proximal adapters
**Inhibitory KIR**		Conserved epitopes on HLA I (“KIR ligands”)	ITIM, Src phosphatases
KIR2DL1	*KIR2DL1*	HLA-C2 alleles (Lys80)	
KIR2DL2	*KIR2DL2*	HLA-C1 alleles (Asp80)*; HLA-C2 alleles (Lys80)	
KIR2DL3	*KIR2DL3*	HLA-C1 alleles (Asp80)	
KIR3DL1	*KIR3DL1*	HLA-B alleles carrying the Bw4 motif*; HLA-A alleles carrying the Bw4 motif	
KIR3DL2	*KIR3DL2*	HLA-A*03, HLA-A*11 carrying specific peptides	
KIR2DL4	*KIR2DL4*	HLA-G	
KIR2DL5		CD155	
**Activating KIR**		HLA-C2, HLA-C1, HLA-F, certain configurations of HLA-peptide combinations	ITAM, SFK
KIR2DS1	*KIR2DS1*	HLA-C2 alleles (Lys80) carrying specific peptides	
KIR2DS2	*KIR2DS2*	HLA-C1 (Asp80) HLA-A*11	
KIR2DS3	*KIR2DS3*	Unknown	
KIR3DS1		HLA-F	
KIR2DS5		Unknown	
Other receptors binding HLA and HLA-like molecules			
ILT2, ILT4 (LILRB1, LILRB2, LIR-1, LIR-2)	*LILR#*	HLA-G	ITIM
NKG2A/CD94	*KLRC1*	HLA-E	ITIM
NKG2C/CD94	*KLRC2*	HLA-E	ITAM, DAP12
NKG2D	*KLRK1*	ULBP1-6, MIC-A, MIC-B	YINM, DAP10
**Death Receptors (Ligands NK cells)**			(in target cell): FADD, Caspase 8
TRAIL-R1, TRAIL-R2, TRAIL-R3, TRAIL-R4, osteoprotegrin	*TNFRSF10A*, *TNFRSF10B, TNFRSF10C*, *TNFRSF10D, TNFSFR11B*	TRAIL	
Fas		FasL	
TNF superfamily members (TNFSF)			
4-1BB (CD137)	*TNFRSF9*	4-1BBL	TRAF1/2 (11)
CD40	*TNFRSF4*	CD40L	TRAF1-6 (12)
**Natural Cytotoxicity Receptors (NCRs)**			ITAM, DAP12
NKp30	*NCR3*	B7-H6, BAT-3, heparan sulfates	
NKp44	*NCR2*	PDGF, heparan sulfates, PCNA	
NKp46	*NCR1*	viral hemagglutinins, heparan sulfates, vimentin, ecto-calreticulin	
**SLAM family receptors**			ITSM, SAP, EAT
SLAMF1 (SLAM, CD150, IPO-3)	*SLAMF1*	SLAMF1	
SLAMF2 (CD48, BLAST-1)	*CD48*	SLAMF4 CD2	
SLAMF3 (CD229, Ly9)	*LY9*	SLAMF3	
SLAMF4 (CD244, 2B4, ERT)	*CD244*	SLAMF2	
SLAMF6 (NTB-A, Ly108, CD352)	*SLAMF6*	SLAMF6	
SLAMF7 (CRACC, CD319)	*SLAMF7*	SLAMF7	
SLAMF8 (BLAME, CD353)	*SLAMF8*	Unknown	
Other receptors involved in NK cell activation and inhibition			
CD16a	*FCGR3A*	antibodies bound to target cells	FcϵRIγ, DAP12, and CD3ζ
DNAM-1	*CD226*	CD155 (PVR)*, CD112	ITAM
TIGIT	*TIGIT*	CD155 (PVR)*, CD112	ITIM
CD96 (TACTILE)	*CD96*	CD155	YXXM and ITIM
TIM-3	*HAVCR2*	Gal-9, Ceacam-1, HMGB1, phosphatidylserine	Tyrosine residues
LAG-3	*LAG3*	MHCII, LSECtin	KIEELE
PD-1	*PDCD1*	PD-L1, PD-L2	ITIM

Cells become targets for NK cell-mediated killing when they acquire DNA mutations, express ligands associated with uncontrolled proliferation, stress, transformation, or are bound by antibodies (4, 18–23). Clinical presentation of a tumor often occurs after transformed cells have evolved mechanisms to escape immune-mediated recognition, and the patient’s NK cells and other immune mechanisms no longer fully control tumor growth (4, 24). Sustained expression of inhibitory checkpoints, shed ligands which block activating receptors, and metabolic dysregulation can impair NK cell-mediated recognition and elimination of tumors (4, 25). Concurrently, tumors can recruit other immune cells that regulate and suppress anti-tumor immune responses, and promote tumor invasion and growth (26) and/or establish physical stromal barriers that can prevent cellular and drug penetration into the tumor’s core (27–30).

NK cells are key effectors against hematologic malignancies, and agents of antibody-dependent cellular cytotoxicity (ADCC) in response to monoclonal antibody therapies (31–33). The presence of NK cells in solid tumors is associated with improved overall survival in a variety of cancers (4, 34, 35) and NK cells contribute to the efficacy of cancer treatment, including an abscopal effect following low-dose radiation, rescue of activity in the presence of immune checkpoint blockade, or activation by chemotherapy (36, 37). Recognizing the anti-cancer potential of NK cells, current clinical trials aim to deliberately support NK cell activation or deliver NK cells expanded *ex vivo* as cellular immunotherapy, including those that employ additional engineering to support strong anti-cancer activity (5, 38–40). In each of these contexts, inflammatory signals, or mechanisms to otherwise strengthen activating signals received by NK cells may tip the balance toward immune-mediated cancer control.

**Figure 1:**
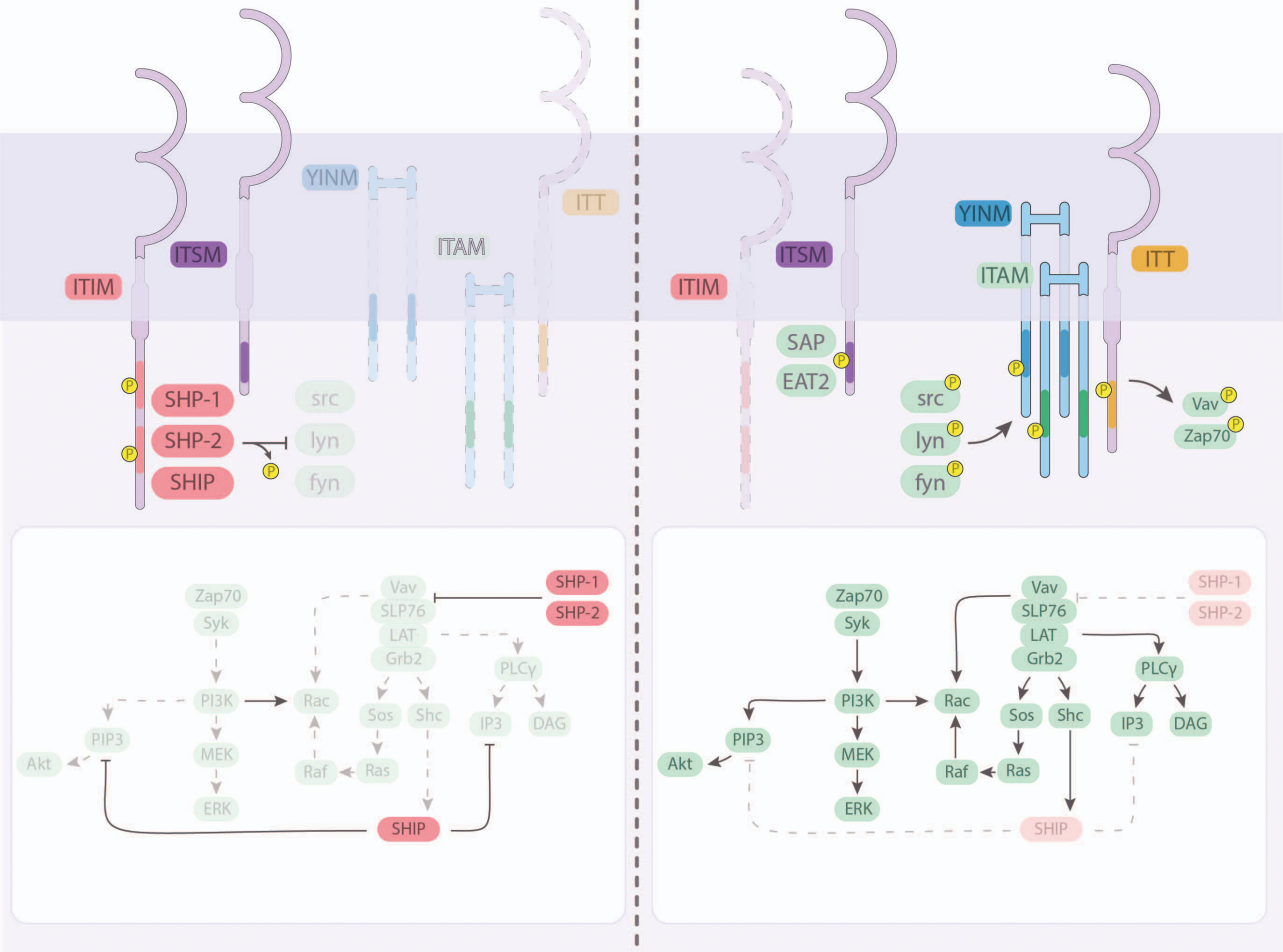
Intracellular signaling and integration downstream of the major NK cell receptors. Most NK cell receptors signal via transmembrane domains, including immunotyrosine-based inhibitory motifs (ITIM), immunoreceptor tyrosine-based switch motifs (ITSM), tyrosine-based signaling motif (YINM), immunotyrosine-based activation motifs (ITAM) and immunoglobulin tail tyrosine (ITT). **(Left)** Inhibitory signals received by NK cells are facilitated by the recruitment and activation of inhibitory SHP-1, SHP-2, and SHIP through ITIM and ITSM. **(Right)** Activating receptor clustering at the immunologic synapse facilitates the activation of intracellular domains by src-family kinases (src, lyn, fyn), or other kinases, including SAP and EAT2. Signals compete to activate or inhibit downstream intermediaries that can lead to transcription factor-mediated cytotoxicity and cytokine production.

NK cell receptors meet target cell ligands in clusters at an immunologic synapse, whose formation is catalyzed by adhesion molecules (primarily ICAM and LFA-1) working in a coordinated effort alongside the reorganization of cytoskeletal components (41, 42). Single receptor-ligand engagement is insufficient to activate the cell and often, concurrent signals for activation and inhibition occur. Synergy between multiple receptors is necessary for cross-phosphorylation of membrane-proximal kinases to initiate a signal cascade that can lead to NK cell activation and degranulation (43). Inside the cell, phosphatases and kinases compete to respond to incoming signals in a dynamic tug-of-war whose outcome can be impacted by environmental features, immunogenetic variation and concurrently-received signals (**Figure 1**) (44).

Recognizing the anti-cancer potential of NK cells, current clinical trials aim to deliberately support NK cell activation or deliver NK cells expanded *ex vivo* as cellular immunotherapy, including those that employ additional engineering to support strong anti-cancer activity (5, 38–40, 45, 46). With engineering, local cytokine support, inflammatory signals, or mechanisms to otherwise strengthen the activating signals received by NK cells may tip the balance toward immune-mediated cancer control. In this review, we discuss the receptor-ligand pairs that govern NK cell interactions with transformed and cancerous cells, their pathways for intracellular signaling, and how alone or in combination, they present opportunities for cancer immunotherapy.

## 2 Receptor driven signaling in natural killer cells

Most interactions between NK cells and neighbouring cells result in non-activation or inhibition, as NK cells survey neighboring, and not necessarily damaged, cells. Provision of inhibitory signals prevents the NK cell exhaustion that might otherwise result from persistent activation, and facilitates maintenance of NK cell education (47). Inhibitory signals are conveyed via engagement of classical and non-classical MHC I molecules, and immune checkpoints (7, 48). Major MHC I-binding receptors include members of the family of killer immunoglobulin-like receptors (KIRs) and the natural killer group 2 (NKG2) family member-A (NKG2A) (49, 50). Other inhibitory receptors may also control NK cell activation, including classical immune checkpoint receptors: TACTILE (CD96), PD-1, TIM-3, LAG-3 and TIGIT (51). Many of these inhibitory receptors convey signaling via immunoreceptor tyrosine-based inhibitory motifs (ITIMs), which, when phosphorylated, recruit phosphatases including the Src homology-containing tyrosine phosphatases (SHP)-1, SHP-2, and SH2 domain-containing inostitol-5-phosphatase (SHIP) (52–54). These phosphatases compete directly with activating signals received by Src-family kinases (SFKs), including Lck, Fyn and Syk (55, 56).

SFKs are key mediators of NK cell activation, dephosphorylated at rest and quickly phosphorylated upon receptor clustering to activate signal intermediaries (57). A subset of KIRs, the Fc receptor CD16a, and the natural cytotoxicity receptors (NCRs) convey activating signals via immunoreceptor tyrosine activating motifs (ITAMs), which activate SFKs, and other signaling intermediaries including ZAP-70 and Vav-1, then phospholipase C, PI3K, Rho-family GTPases (58, 59). Additional activating receptors include the NKG2 family members NKG2C and NKG2D, which signal via DAP12 and DAP10, respectively, and therefore bypass the need for SFKs, instead shunting directly to activating downstream mediators including Vav-1, PI3K, Rho-family GTPases and phospholipase C (60–63). Immunoglobulin tail tyrosine motifs (ITTs), used by DNAM-1, are phosphorylated by SFKs and similarly drive downstream activation (64, 65). Hence, although they are initiated separately, multiple activating pathways converge on SFKs, so their signaling can be additive.

Immunotyrosine-based switch motifs (ITSMs), encoded in SLAM-family receptors and PD-1, combine the features of ITAMs and ITIMs by associating with SH2 domain-containing proteins, including SHP-1, SHP-2, and SHIP-1, (which convey inhibitory signaling), and SLAM-associated protein (SAP) and Ewing’s sarcoma-activated transcript 2 (EAT2), which support activation (66). Activation signals proceed via SFKs. Inhibitory SH2 domain-containing proteins prevent these signals in two ways: directly, by occupying the docking site on the ITSM, and indirectly, by the phosphatase activity of SHIP, SHP-1 and SHP-2 (66). In this way, one ITSM-containing receptor can switch between signaling for inhibition or activation.

The outcome of concurrent activating and inhibitory signals result in adjustments to the levels of transcription factors, including NF-κB, which mediate NK cell activation-related genes (67). These genes regulate NK cell functions such as cytotoxicity, microtubule organization and granule polarization (67). In some cases, NK cells are induced to become “adaptive” or memory-like NK cells: a state driven by epigenetic changes where NK cells are noted for their long lifespans, ability to recall previous challenges, and rapid, potent responses (68–70).

The redundancy of the intracellular signaling cascades downstream of the major NK cell inhibitory and activating receptors enables crosstalk and integration of signals. Hence, with combined and simultaneous signalling from different receptors, each NK cell can balance and calibrate an appropriate response.

### 2.1 Human leukocyte antigen class I: a major ligand for NK cell receptors and NK cell education

Human leukocyte antigens (HLA) are the most polymorphic gene in the human genome, and expressed on all healthy nucleated cells at variable densities that reflect cellular health and HLA allelic diversity (71, 72). HLA-A, HLA-B and HLA-C are together classified as “conventional HLA I”, or class Ia molecules, and represent the most polymorphic HLA I alleles (72). Unconventional HLA I molecules, classified as class Ib, include HLA-E, HLA-F, and HLA-G (71). These exhibit relatively low polymorphism, and have important roles in tolerance, inhibition, and pregnancy, with HLA-E remaining relatively conserved between human and primates (72–76). Each HLA I, except for HLA-F, is comprised of three alpha domains, and stabilized by β2 microglobulin and by peptides presented in the binding groove of its variable domain (57). HLA-F is less understood, but can associate with β2 microglobulin and exhibits at least two configurations: closed, where it seems to not present peptides, and open, where it presents uncommonly long peptides in an open-ended binding groove (77).

**Figure 2:**
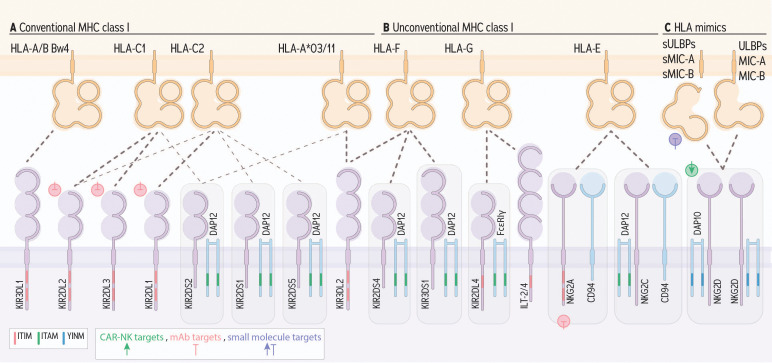
NK cell receptors engaging HLA I and conveying activating or inhibitory signals. KIRs engage conserved epitopes on groups of HLA I molecules. **(A)** KIR receptors with long (L) cytoplasmic tails generally convey signals for inhibition via an ITIM, except for KIR2DL4. **(B)** KIR receptors with short (S) cytoplasmic tails and KIR2DL4 convey signals for activation by engaging DAP12, or FcϵRIγ, respectively, which contain ITAMs. Beyond KIR, ILT-2 and 4, and NKG2A (heterodimerized with CD94) bind with MHC I and convey inhibitory signals via ITIMs. NKG2C (heterodimerized with CD94) and NKG2D engage with HLA-E or HLA orthologs which include ULBPs, MIC-A and MIC-B. **(C)** Finally, NKG2C associates with DAP12 and NKG2D signals via DAP10, a YNIM-containing receptor, each to signal for activation. Green, pink, and purple symbols indicate CAR-NK targets, mAb targets, and small molecule targets, respectively.

HLA I is co-evolving with the killer immunoglobulin-like receptors (KIR) and members of the NKG2 family (78). Their interactions can signal for activation or inhibition, depending on the specific receptors ligated, bound peptides, immunologic experience, and compound allelic partnerships (3, 79) (**Figure 2A, B**). Groups of conventional HLA I molecules display conserved public epitopes that enable groupings of HLA I molecules as “KIR ligands”: HLA-C molecules expressing Asp or Lys at position 80 define the HLA-C1 or C2 subgroups, respectively, and account for all HLA-C molecules (80, 81). A subset of HLA-A and B molecules each contain the HLA-Bw4 motif, and some specific alleles of HLA-A (HLA-A*03 and *11) can act as NK cell ligands, and the balance of HLA-A and -B alleles are not known to engage with any KIR (79, 82, 83).

KIR molecules with long cytoplasmic tails signal for inhibition via ITIMs encoded in their transmembrane regions. HLA-C1 engages **KIR2DL3** and HLA-C2 engages **KIR2DL1** (84). **KIR2DL2** preferentially binds with HLA-C1 molecules, but some alleles also exhibit binding affinity with HLA-C2 (85, 86). HLAs harbouring the Bw4 motif bind with **KIR3DL1**; the remainder of HLA-B molecules and most of the HLA-A molecules are not known to encode a ligand for KIR (87). **KIR3DL2** binds to HLA-A*03 and A*11 molecules, but only when they present certain peptides, including exogenous CpG DNA and peptides derived from Epstein-Barr virus (82, 83, 88). Inhibitory **KIR2DL4** and immunoglobulin-like transcript **(ILT)-2 and -4** molecules on NK cells bind HLA-G and signal via ITIM (89). HLA-G is an unconventional HLA molecule whose expression is normally limited to immune privileged sites including the cornea (90) and placenta (91), but has also been found upregulated, and associated with poorer prognosis in several cancers, including colorectal cancer (92), pancreatic cancer (93), breast cancer (94), and ovarian cancer (95). Soluble HLA-G has been found to dampen NK cell function, including impairment of NK cell migration to inflamed tissues (96).

KIR molecules with a short cytoplasmic tail recruit the ITAM-containing molecule DAP12 to the immunologic synapse. Activating KIR, including **KIR2DS1**, **KIR2DS2**, and **KIR3DS1** are each known to bind with HLA I molecules, albeit with lower affinity than their inhibitory counterparts (97, 98). Additional activating KIRs (KIR2DS3, KIR2DS5) also have ITAMs and may contribute to activation and may bind with specific alleles (99), though the impact of these ligands and their corresponding signals have not been well established.

KIR2DS1 binds HLA-C2 (Lys^80^) and, at high levels, individuals that are homozygous for HLA-C2 have KIR2DS1+ NK cells that are hyporesponsive (97, 100, 101). This has implications in hematopoietic cell transplantation, where HLA-matching is only beneficial in the absence of homozygous HLA-C2 (102, 103). Moreover, for the treatment of B-cell malignancies, addition of rituximab to chemotherapy did not improve survival outcomes in patients with KIR2DS1-HLA-C2/C2 (104), suggesting that KIR2DS1 and HLA-C status could be a predictive marker for efficacy of rituximab due to the potential of hyporesponsive NK cells (104).

Although KIR are not known to recognize HLA-presented peptides specifically, the biochemical features and structural/allosteric variations that they introduce can influence receptor binding and consequent signaling (57, 105). This is the case for KIR2DS2, which binds conditionally to HLA-C1 and HLA-A*11 alleles, when they present viral peptides, including those derived from viral helicases (3, 106), though their role in cancer is unclear. Similarly, KIR3DS1 is activated specifically by the open (peptide presenting) configuration of HLA-F (107).

The NKG2 family members engage with HLA-E, but in a manner distinct from conventional KIR-HLA interactions (108). **NKG2A**, an ITIM-containing receptor, and **NKG2C**, which engages ITAM-containing DAP12 for signaling, both bind with HLA-E and heterodimerize with CD94 (49, 50, 109–111). HLA-E molecules present the leader peptides of conventional HLA molecules but also present antigenic peptides, including neoantigens, to both T and NK cells (105). In healthy tissues, HLA-E expression is low (112), but high HLA-E expression is linked to poor patient outcomes in several cancer types, including gastric cancer (113, 114), colorectal cancer (113), pancreatic cancer (93), breast cancer (94), renal cell carcinoma (115), ovarian cancer (116), and glioma (111, 117), suggesting a dominant impact of NK inhibition on limiting anti-cancer activity. Indeed, high expression of NKG2A, both alone and with high HLA-E expression, is associated with poorer patient survival in liver cancer (118). Likewise, co-expression of the high-density HLA-E allele (HLA-E*01:03) and NKG2A associates with Epstein-Barr virus-associated lymphoma (119). Given the known impacts of HLA-E expression on cancer development, it is likely that these are exacerbated by NKG2A expression on NK cells in other cancer types, though this remains to be studied extensively.

In addition to signaling during target cell engagement, KIR-HLA and NKG2A:HLA-E interactions are central to a process called NK cell “education”, “tuning” or “licensing” (120–122). Each of KIR and NKG2A are expressed on only a subset of NK cells, their HLA ligands may or may not be available in the host, and the avidity of receptor-ligand binding is variable. Therefore, the extent to which each NK cell can be inhibited by “self” HLA varies. An individual’s NK cell repertoire therefore consists of both educated and uneducated cells (85, 123). Resultantly, the reactive thresholds of individual NK cells differ, and create a spectrum of responsiveness and anti-tumor effector function that varies within and between individuals (3, 122, 124).

NK cells that are most sensitive to inhibition by “self” HLA I exhibit a greater mobilization of activating receptors in the actin meshwork (8), greater DNAM-1 expression (125), a greater density of granzyme B (9) and lower levels of SHP-1 (53)– making them more easily activated in the absence of strong inhibition. Sensitivity to inhibition, however, is the Achille’s heel of educated NK cells in cancer therapy because HLA I on tumors often persists, or becomes upregulated in response to IFN-γ (126). Strategic selection of KIR and HLA allelic combinations and NK education status, or blocking signals for inhibition, will therefore be critically important for the success of NK cell-based immunotherapy (3, 124).

During cancer development, there is extensive pressure on HLA expression driven by immune activity: T cell-mediated recognition may select for clones lacking HLA expression, but this may create a target for NK cells by interrupting signals for inhibition. Downregulation of classical HLA I expression is reported for several solid cancers, including melanoma, cervical, breast, colorectal and lung cancers (127–130). HLA I loss may represent partial or complete losses of HLA gene loci (129, 131), or components of the HLA processing and presentation pathway (127, 131). Frequently, loss of heterozygosity for the *HLA-ABC* genes, and other genes involved in HLA I processing and presentation, is observed following acquired resistance to immune checkpoint inhibitors like anti-PD1/PD-L1 (127, 132, 133).

*KIR* is highly polymorphic; an individual’s KIR configuration has been associated with both the risk of developing cancer and their ability to respond to cancer therapies. KIR expression is not known to change as a function of cancer, but the presence of self-sensitive KIR can enable inhibition of otherwise-activated NK cells. Beyond strategic donor selection to optimize KIR/HLA genotype for potent NK cell alloreactivity, both KIR and NKG2A can be directly targeted through monoclonal antibodies. Lirilumab, an anti-KIR2DL1/2/3, has been tested in a phase I clinical trial against multiple myeloma, and supports alloreactivity of NK cells (134). Monalizumab (anti-NKG2A), in combination with Cetuximab, has shown anti-tumor effects in a clinical trial of patients with squamous cell carcinoma of head and neck with high expression of HLA-E and NK cell infiltration. Taken together, this implies the important role of selecting/regulating KIR/NKG2A in cancer treatment.

### 2.2 NKG2D and stress ligands

Perhaps the best known member of the NKG2 family, **NKG2D**, is unique in that it forms homodimers (135), and signals for activation via DAP10 (62). Ligands for NKG2D are structural homologs that co-evolved alongside HLA I molecules and include MHC class I chain-related molecule A and B (MICA/B), and UL16 binding proteins 1,2,3,4,5 and 6 (ULBP1-6) (**Figure 2C**) (136, 137). NKG2D ligands are typically expressed at low cell surface densities, but their density on the cell surface increases in response to DNA damage (138), oncogene activation (138), infection, excessive proliferation, and oxidative stress, earning them the title of “stress ligands” (138). The NKG2D receptor itself is responsive to cytokines associated with immune priming (ie. IL-2, IL-15), which increase basal phosphorylation of DAP10, priming the cell to deliver activating signals (139). Together, NKG2D and its ligands generate a robust system to detect hallmark features of stressed cells.

Presented on a target cell, NKG2D ligands signal for NK cell activation, and NKG2D is central in cancer immunosurveillance. In mouse models, antibody-mediated neutralization of NKG2D interrupted immunosurveillance that otherwise prevented carcinogen-induced tumors (140). Likewise, elimination of NKG2D using microRNA silencing rendered mice more susceptible to cancer growth (140–142). In humans, histological studies have confirmed high and co-expression of NKG2D ligands in cancers of the breast (142, 143), colon (144, 145), gastric system (146, 147), lung (148, 149), skin (150), ovary (151, 152), pancreas (153), prostate (148, 154), and kidney (148). For NKG2D, diminished receptor expression is known to occur in response to hypoxia and diminished STAT3 activity (155), and in response to shed soluble ligands released following protease-mediated cleavage, which block the receptors from signaling (156).

Existing and nascent approaches to cancer therapy alter the expression of NKG2D ligands and encourage NK cell-mediated tumor killing. For example, ionizing radiation (157, 158), histone deacetylase (HDAC) inhibitors (159), and chemotherapy (158, 160) each prompt increased levels of NKG2D ligands. Furthermore, agents that prevent ligand matrix metalloproteinase activity by small molecule inhibitors can potentiate NK cell response by preventing ligand shedding (161).

The NKG2D receptor is a logical target for immunotherapy, and it is now being incorporated into immunotherapeutic approaches including NKG2D-CAR-NK, NKG2D-CAR-T cells (162, 163), CAR-T cells with a DAP10 intracellular domain (164), and bi-specific killer-engagers (BiKEs) (165, 166). While these therapies have indeed enhanced NK cell mediated anti-tumor activity, complete tumor control will require combination therapies that extend beyond the targeting of NKG2D alone.

### 2.3 TNF- receptor superfamily ligands

**Figure 3:**
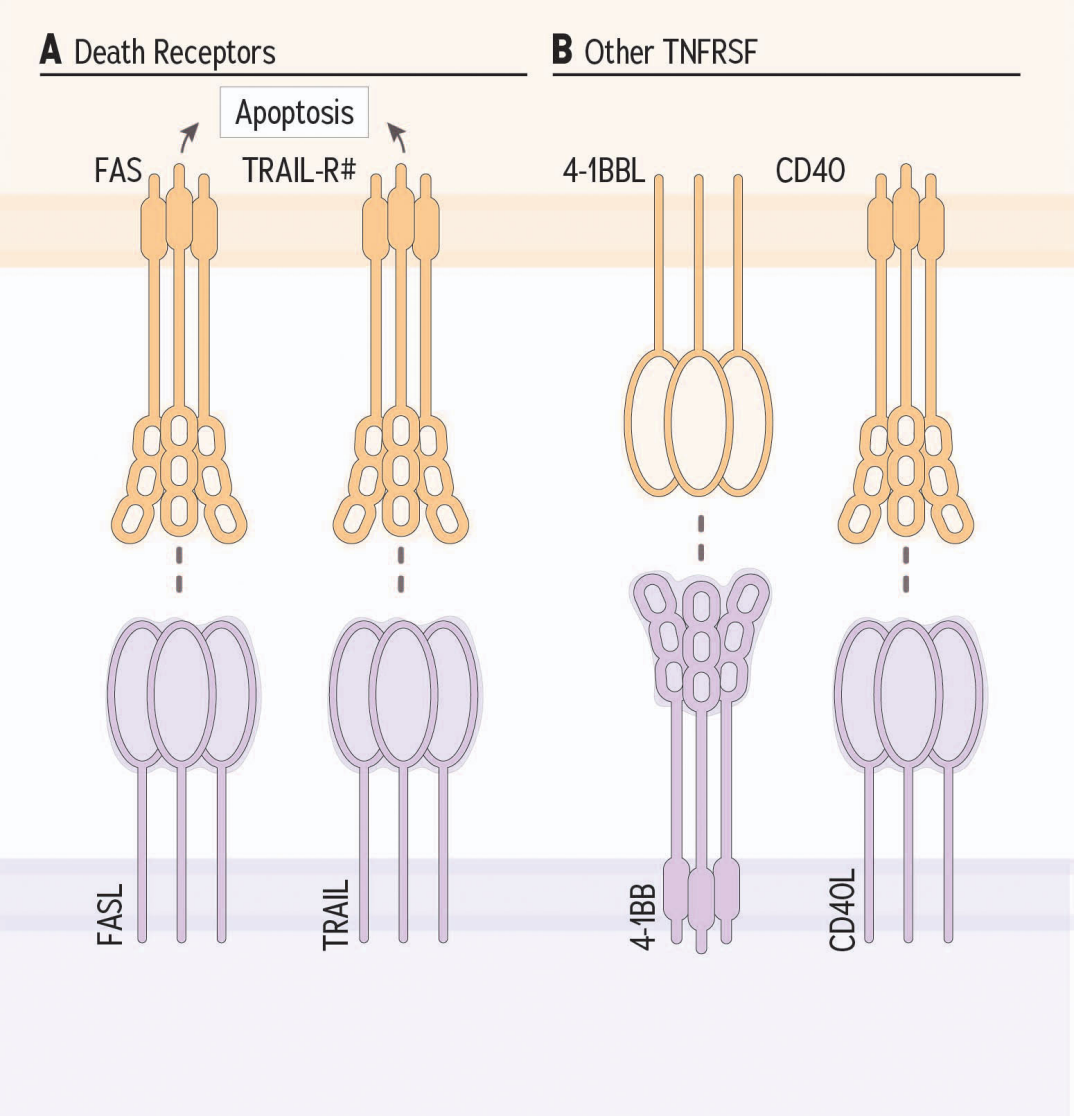
TNF receptor superfamily receptor family member receptor and ligand partnerships and NK cells. **(A)** Death receptors expressed by target cells are induced for apoptosis when their ligand is provided by NK and other cellular sources. **(B)** Costimulatory TNFRSF members, 4-1BB and CD40L potentiate NK cell response against target cells.

The tumor necrosis factor receptor superfamily (TNFRSF) is a group of proteins that primarily regulate cell activation, differentiation, and survival, either as membrane-bound factors or cleaved, soluble factors. Here, we focus our discussion on those studied for their roles in NK cell function and reactivity, including the death receptor ligands Fas and TNF-related apoptosis inducing ligand (TRAIL), and costimulatory members 4-1BB and CD40L (**Figure 3**). TNFRSFs engage TNF superfamily (TNFSF) ligands, which can often be in membrane bound and soluble forms (167). This family of receptors and ligands signal primarily through NF-κB to induce proinflammatory function, or induce apoptosis of the target cell (168). Notably, while other TNFSF members have been reported to be expressed by NK cells, or to impact their function [i.e. GITR (CD357) (169), LIGHT (CD258) (170) and CD70 (171)], relatively little is known on their roles and targetability in cancer; these remain open questions and opportunities.

#### 2.3.1 Death receptor ligands (TRAIL and FasL)

“Death receptors” include Fas and TNF-related apoptosis inducing ligand (TRAIL) receptor; they are present on putative target cells and facilitate the induction of apoptosis when bound by death receptor ligands (172–174) (**Figure 3A**). The typical role for these receptor-ligand partnerships is to facilitate normal cell turnover and immunoediting; for instance, to remove aged or damaged cells, or reduce an expanded population of effector cells once an infection is cleared (175). Each of **FasL** and (**TRAIL**) can be provided by NK cells, and exist as membrane-bound or soluble ligands (176). The role of soluble TRAIL is not well understood and only membrane-bound FasL, not soluble FasL, is capable of inducing target cell apoptosis (177).

FasL has a single known receptor: Fas, and TRAIL has multiple receptors: TRAIL-R1, TRAIL-R2, TRAIL-R3, TRAIL-R4 and the soluble decoy receptor osteoprotegrin (OPG) (178). Fas, TRAIL-R1, and TRAIL-R2 each contain a death domain that bind Fas-Associated Death Domain (FADD) adaptor, which recruits the Death Inducing Signaling Complex (DISC) to initiate caspase-8 activation and apoptosis (179, 180). Fas and TRAIL receptor signalling can also initiate activation of NF-κB which, in contrast to the pro-apoptotic signaling generated by the death receptors, can paradoxically contribute to target cell survival and proliferation (181, 182). For this reason, TRAIL-R3, TRAIL-R4 and OPG, which lack a death domain and have been classified as decoy receptors, may contribute to cancer cell survival and proliferation (183). Targeting apoptosis, but not cellular activation, will therefore be critical in therapies that aim to leverage death receptor signaling for cancer killing.

Agonists have been developed to target TRAIL receptors on cancer cells; they have been demonstrated as safe, but relatively ineffective as monotherapy [reviewed in Snajdauf et al., 2021 (184)]. For example, mapatumumab and conatumumab, TRAIL-R1 agonistic antibodies are well-tolerated, but ineffective as monotherapy to patients with solid tumors (185–188). However, stable disease was enabled in the presence of mapatuxumab combined with apoptosis-inducing chemotherapy, or the tyrosine kinase inhibitor sorafenib (184, 189), highlighting how the simultaneous targeting of multiple death pathways might prevent tumor escape. Similar agonistic approaches may be possible for targeting Fas, but preclinical models have revealed a potential unexpected benefit of blocking Fas: survival of tumor-infiltrating T cells, which may, in turn, enhance the efficacy of immunologic checkpoint blockade (190). Altogether, the available, but limited studies, reveal a potential role for death receptor signaling in cancer immunotherapy, but little is known specifically of the roles of NK cells in these pathways.

#### 2.3.2 Costimulatory TNFSF members

Costimulatory molecules of the TNFSF are best studied for their roles in T cells, and although many can also impact NK cell function, relatively few experiments have explored this. Here, we highlight two TNFSF members with known costimulatory function in NK cells, acknowledging that further research is required to classify whether additional TNFSF members are expressed, functional and relevant on NK cells (**Figure 3B**).

**CD137 (4-1BB)** is a transmembrane glycoprotein expressed on NK cells that engages 4-1BBL during NK cell activation to enhance cytotoxicity and expansion by increasing the expression of effector molecules including granzyme B, perforin and FasL (191–193). These functional enhancements are a result of increased MAPK signaling resulting in NF-κB activation within NK cells (193). The inclusion of 4-1BBL on feeder cells (in combination with IL-21) is used in expansion of NK cells for clinical trials (190). Addition of 4-1BBL promotes upregulation of activating receptors including the NCRs, CD16, and SLAM family members, alongside inhibitory KIR, all via STAT3 activation (194).

CD40 ligand **(CD40L**, CD154**)** is best understood as a ligand upregulated on T cells and macrophages, but can be upregulated on NK cells in response to IL-2 stimulation and during NK cell expansion (195–197). The significance of CD40L on NK cells remains to be investigated, but when CD40L is provided by T cells, the result is signals for activation and maturation in antigen presenting cells. Indeed, in mouse models, depletion of NK cells and blockade of CD40L had a similar effect on antigen presentation in the tumor draining lymph nodes: both resulted in lower cytotoxic T cell priming, but whether CD40L is obligately provided by NK cells was not studied (198). Nevertheless, these, and other co-stimulatory TNFSFR members expressed on NK cells could represent good candidates for immunotherapy.

### 2.4 Natural cytotoxicity triggering receptors

**Figure 4:**
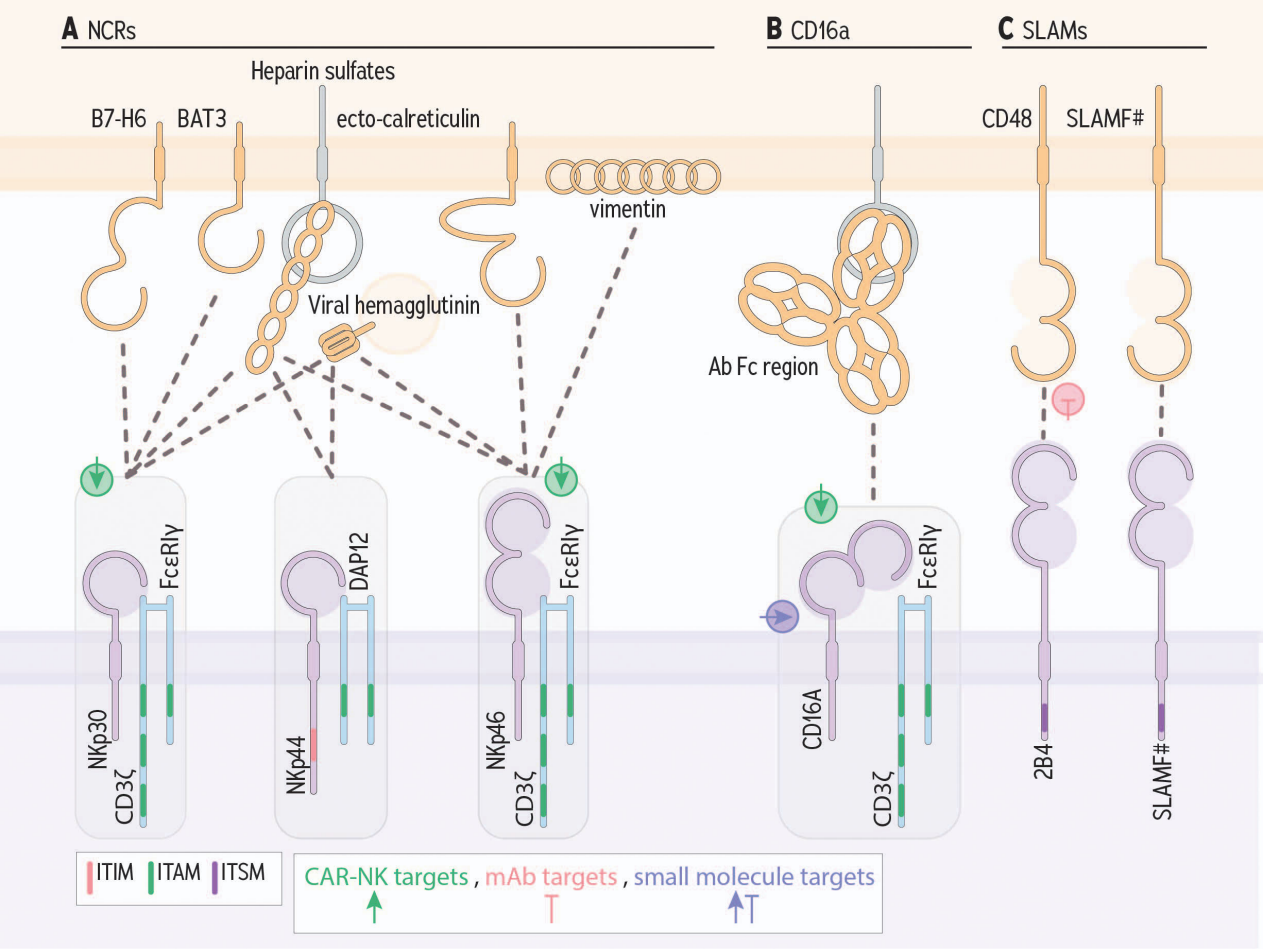
Germline encoded NK cell receptors and ligands that contribute to cancer cell killing. **(A)** Natural cytotoxicity receptors bind an array of ligands; shown are the most important to tumor cell recognition. **(B)** CD16a, binds the constant region/fragment crystallizable (Fc), portion of antibodies that, when bound to target cells, enable cross linking of receptors. **(C)** SLAM family members can signal for activation or inhibition via an immunotyrosine switch motif. Green, pink, and purple symbols indicate CAR-NK targets, mAb targets, and small molecule targets, respectively.

The **natural cytotoxicity triggering receptors (NCRs)** are a group of HLA III genes that primarily generate activating signals in response to ligand binding through a transmembrane-encoded ITAM, or association with ITAM-containing adapter molecules (199) (**Figure 4A**). The NCRs do not themselves encode ITAMs, and instead recruit and signal through ITAM adapter molecules, including DAP12, CD3ζ and FcϵRIγ (199). Each exist as splice variants, with some isoforms conveying inhibitory signals via an ITIM-like sequence (200). Although there are others, three NCRs that have been studied in the context of cancer are NKp30, NKp44, and NKp46.

**NCR ligands** typically become available as a result of cellular stress, inflammation, and transformation (199). They are often expressed on tumor cells, or released during oncogenesis, and likely evolved to respond to ligands available in disease contexts. Cancer-derived ligands are not the exclusive binding partners for the NCRs, but for simplicity, we focus on those involved in cancer pathology here.

- **B7-H6** is a B7 costimulatory family member that is overexpressed and associated with poorer outcomes in cancer (201, 202). B7-H6 and BAT-3 can be shed from the membrane and antagonize NCR binding to prevent NK cell activation (203).
- **BAT-3** normally contributes to stabilizing p53 and contributing to tumor suppression (204). It may also be released in exosomes derived from cancer cells; in this configuration they can drive NK cell activation against cells that have lost normal p53 function (205).
- **Heparin sulfates** are components of the extracellular matrix that are exposed during cellular migration and tumor metastasis (206).
- **Platelet-derived growth factor (PDGF)** is involved in angiogenesis and cellular proliferation and is frequently expressed by tumor cells (207–209). There are at least five dimeric isoforms; among them, PDGF-DD is established as the ligand for NKp44 [reviewed in (199)].
- **Proliferating cellular nuclear antigen (PCNA)** is involved in DNA replication, repair, and remodelling (210). Among the ligands for NKp44, PCNA is the only one known to generate inhibitory signals. Evidence supports a protective immunoregulatory role in pregnancy, and the overexpression of PCNA by tumors may enable escape from NK-mediated immunosurveillance (211).
- **Vimentins** are structural components of mesenchymal cells, and they have been used as a biomarker of epithelial-to-mesenchymal transitioning, which is associated with metastasis, in an array of solid tumors (212, 213).
- **Ecto-calreticulin** is externalized calreticulin, which is typically released from the endoplasmic reticulum in response to stress. Most notably, this occurs in response to chemotherapy-induced cell death and senescence (18).

**NKp30** binds B7-H6 (205), BAT-3 (205), and heparin sulfates (214, 215). NKp30’s transmembrane domain associates with the ITAM-containing adaptors CD3ζ and FcϵRIγ. There are at least six splice variants of NKp30, which differ in their tissue distribution, engagement with adapter molecules, and outcomes upon ligand binding, notably on production of IFN-γ and IL-10 (216). Variants a-c are the most common, with a and b favouring IFN-γ production and NKp30c associating with IL-10 production (216). B7-H6 has been proposed as a biomarker for development and progression in an array of cancers (217–219); by extension, leveraging this expression as a target for immunotherapy may be possible.

**NKp44** signals for activation when bound by PDGF and heparan sulfates, and signals for inhibition upon binding to PCNA (199, 215, 220, 221). Three splice variants are described for NKp44, where the transmembrane domain in the NKp44b and NKp44c isoforms associate with DAP12 to signal for NK cell activation (222). NKp44a signals via an ITIM-like domain and binds PCNA, and at high surface densities, this splice isoform can inhibit NK cell function (200, 223). Dominant expression of this “inhibitory” NKp44 isoform is associated with poorer survival in patients with acute myelogenous leukemia (223), and PCNA is currently being explored as a biomarker and target for monoclonal antibodies (224).

**NKp46** associates with CD3ζ and FcεRΙγ (225, 226). Key cancer associated ligands for NKp46 include heparan sulfates (215), vimentin (199, 225), and ecto-calreticulin (18). NKp46 expression is correlated with the degree of NK cell response generated (227) and IFN-γ produced via NKp46 signaling alters the deposition of fibronectin limiting metastasis in murine melanoma (228). Like NKp30, the ligands for NKp46 have been proposed as biomarkers for cancer severity and progression (229), endorsing these as potential targets for NK cell-based immunotherapies.

Targeting NCRs or their ligands for cancer immunotherapy is an area of active exploration that warrants investigation in NK cell-based cancer immunotherapy. Current studies are investigating the potential of using BiKEs or CARs that use the NCRs to enhance tumor recognition (230, 231). For example, a trifunctional natural killer cell engager (TriKEs) targeting the AML antigen, CD123, while simultaneously binding NKp46 and CD16a on NK cells has demonstrated efficacy in murine models and nonhuman primates (232) and is currently being tested in early clinical trials (NCT05086315). A similar strategy – coupling NKp46 engagement to anti-CD20, CD16 and the IL-2R beta chain has likewise generated promising preclinical results (233). NKp30 has been targeted on tri-specific engagers, coupling NKp30 engagers with Fab and anti-EGFR to create a potent pathway for tumor cell lysis and NK cell cytokine production (234). These strategies are in their infancy, but illustrate the promise of targeting such conserved NK cell ligands for immunotherapeutic purposes.

### 2.5 CD16a and antibodies

**CD16a** is expressed on NK cells, monocytes, and macrophages, and is the major receptor engaged for ADCC (**Figure 4B**) (235). CD16a encodes an ITAM motif, which recruits the signaling adapters FcϵRIγ, and CD3ζ to signal NK cells for proliferation, survival, cytokine production and degranulation (43, 236). Notably, CD16a activates NK cell degranulation, even in the absence of other activating signals (237).

Antibodies can function by neutralizing targets or opsonizing them for phagocytosis or killing. Target cell binding by antibodies enables cross-linking of CD16a; these antibodies can be produced endogenously by plasma cells or delivered as therapy (21). In NK cells, CD16a Fc receptor crosslinking triggers ADCC (238). NK cell-mediated ADCC is a central mechanism to the killing primed by anti-HER2 (trastuzumab) (239) anti-GD2, (dinutuximab) (240), and anti-CD20 (rituximab) (241). In mice deficient for CD16a, or in which CD16a engagement with antibodies is blocked, tumor growth is exacerbated (239). Noteworthy, CD16a is susceptible to cleavage by metalloproteases within the tumor environment, and these soluble fragments could block CD16a on NK cells and create an additional opportunity to evade ADCC (242). Strategies to strengthen antibody binding and Fc receptor signaling are being explored and include antibody Fc engineering to maximize binding and activation, non-cleavable CD16 molecules, bi-specific antibodies, and antibody-drug conjugates (37, 238, 243).

### 2.6 SLAM receptors

**Signalling lymphocytic activating molecule (SLAM)-family receptors** are a group of type I transmembrane receptors, expressed on hematopoietic cells (244), especially during and shortly after differentiation (**Figure 4C**) (245). SLAM-family receptors expressed on NK cells include SLAMF1, SLAMF2 (CD48), SLAMF3, SLAMF4 (2B4), SLAMF6, SLAMF7 (CRACC) and SLAMF8– with CD48, 2B4, and CRACC being the most prominent (246).

These SLAM-family receptors, excluding CD48 and SLAMF8, signal through ITSMs (247). SLAM-family receptors are homotypic, except for the partnership of 2B4 and CD48 (248, 249). CD48 and 2B4 also, uniquely, bind in both *trans* (ie. with other ligand-expressing cells) or in *cis* (ie. on the NK cell surface) (250, 251). *Cis* binding conveys baseline 2B4 ITSM phosphorylation, and a higher signalling threshold for activation due to competition with ligands for binding in *trans* (248, 250, 251).

SLAM-family receptors can be expressed on hematologic (252) and solid tumors (245, 249). Indeed, CD48-2B4 signalling has been linked to early NK cell activation by monocytes, followed by exhaustion (253). NK cell monocytes isolated from hepatocellular carinoma highly express CD48, and blocking this CD48-2B4 interaction leads to relatively decreased NK cell activation and sequential exhaustion (253). Monocytes isolated from hepatocellular carcinoma have high CD48 expression and blocking the 2B4-CD48 interaction decreased NK cell activation and exhaustion (253). This provides evidence that a multitarget approach might be necessary for checkpoint therapy, and that targeting SLAM-family receptors (whether inhibiting or activating these receptors) is not a one-size-fits all for each tumor. Additionally, both CD48 and CRACC are highly expressed on multiple myeloma, and these are now being investigated as targets for monoclonal antibody therapies (254). Monoclonal CD48 has shown promising pre-clinical results at decreading multiple myeloma tumor growth (252). Undoubtedly, the extensive expression of SLAM family members and their ability to contribute to NK cell activation will promote further investigation toward their clinical use.

### 2.7 DNAM-1, TIGIT and CD96

**Figure 5:**
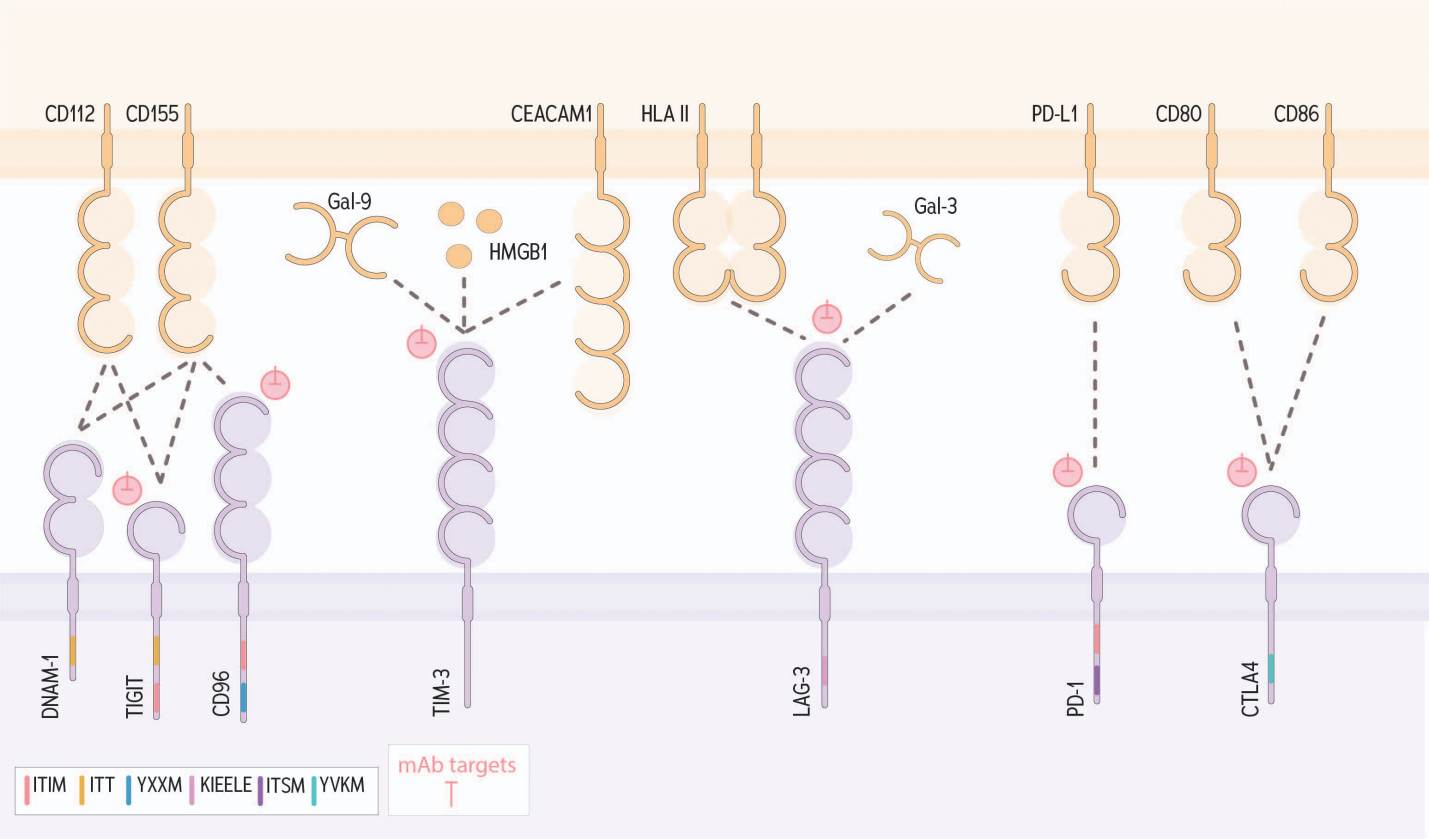
DNAM-1, TIGIT, CD96 and other immunologic checkpoints that contribute to NK cell regulation within a tumor. The adhesion molecule CD112 is recognized by both DNAM-1 and TIGIT. Engagement with DNAM-1 results in activation via ITT signaling, conversely binding to TIGIT results in net inhibition signed through an ITIM. CD155, another adhesion molecule, binds to DNAM-1, TIGIT and CD96; signaling through CD96 through the signaling motifs tyrosine-based sorting motif (YXXM) and ITIM. TIM-3 can bind a variety of ligands including Gal-9, HMGB1 and CEACM1 to signal for inhibition. LAG-3 is known to bind HLA II and Gal-3. PD-1 binds PD-L1 and CTLA4 binds to CD80/CD86 to signal for inhibition. Pink symbols indicate mAb targets.

**The DNAX-accessory molecule-1 (DNAM-1)** and **TIGIT** receptors are commonly expressed on NK cells and T cells, and to interact with the same ligands: CD155 and CD112 (64, 255, 256). DNAM-1 is an activating receptor that signals via ITT; TIGIT is an inhibitory immune checkpoint that encodes an ITIM (65, 257) (**Figure 5**). **CD96 (TACTILE)** also contains an ITIM, and binds to CD155, but not CD112 (257), and is thought to have secondary roles in cell-cell adhesion (257). Recently, KIR2DL5, an ITIM-containing KIR, has also been identified as a receptor for CD155 (258).

Each of CD155 and CD112 bind TIGIT and DNAM-1, signaling for inhibition through TIGIT or activation through DNAM-1. CD155 and CD112 both bind TIGIT with a higher affinity that DNAM-1 (259, 260). CD155 and CD112 are expressed at low levels on healthy cells and are upregulated in response to inflammation, cellular stress, reactive oxygen and nitrogen species (261–263), and following chemotherapy (264). Overexpression of these ligands on the tumor is usually associated with poorer prognosis for patients: increased tumor expression of CD112 correlates with increased tumor size and stage, in cancers of the gallbladder (265), colon (266), ovary (267), and pancreas (268). Likewise, tumor overexpression of CD155 is associated with poor prognosis and progression of multiple cancers (269) including breast cancer (270), gastric cancer (271), non-small cell lung cancer (272), melanoma (273), colorectal cancer (274), and sarcoma (275).

TIGIT modulates DNAM-1 mediated activation through the competitive binding of their shared ligands (276). Further, TIGIT directly inhibits DNAM-1 activation through *cis* interactions that interfere with DNAM-1 homodimerization (277). TIGIT blockade enhances NK cell responsiveness both *in vitro* and *in vivo*, particularly when DNAM-1/CD155 interactions remain intact (277, 278). In mouse models, blockade of CD96/CD155 binding results in reduced metastatic spread *in vivo–* in part by enabling DNAM-1/CD155 binding through loss of CD96 competitive binding (279, 280).

Through alternative splicing, CD155 can be released in a soluble form (sCD155), which binds to DNAM-1 with greater affinity than TIGIT or membrane-bound CD155, and block binding and signaling for activation via DNAM-1 (281). High expression of sCD155 is associated with poor prognosis and increased cancer progression in lung, breast, liver, and gynecological cancers (282–284). sCD155 binding can also result in the endocytosis of DNAM-1, making it less available to generate signals for activation (285, 286).

The opposing roles these receptors play is evident in cancer progression; in acute myeloid leukemia, high expression of DNAM-1 is associated with longer progression-free survival and overall survival (287), while increased TIGIT expression was correlated with NK cell dysfunction and poorer patient outcomes (288). Further, CD96 expression was linked with an increased immunosuppressive immune signature and poor patient prognosis in gastric cancer (289).

Targeting TIGIT, and thereby blocking its inhibitory interaction with CD155 and/or CD112, has been the focus of recent research, with several monoclonal antibodies currently undergoing clinical trial. A phase II trial assessing tiragolumab in combination with atezolizumab demonstrated an objective response rate of 37%, with an objective response rate of 66% in patients with a high PD-L1 status. However, some adverse events were observed, with two treatment-related patient deaths (290). Phase III trials are currently under way for both small cell and non-small cell lung carcinoma (NCT04256421; NCT04294810), as well as trials of tiragolumab against other malignancies, such as melanoma (NCT05116202; NCT03554083), pancreatic cancer (NCT03193190), gastric cancer (NCT0493322; NCT05251948), and cervical cancer (NCT04300647). Of course, administration of these antibodies can impact other lymphocytes, and understanding the specific roles of NK cells compared with other lymphocytes are needed.

### 2.8 Immunologic checkpoints primarily known for T cell impacts: TIM-3, LAG-3, PD-1

Immunologic checkpoints are best studied in the context of T cells in cancer but have similar functions in NK cells (51). Immunologic checkpoints known to impact T cells may also inhibit NK cells. These include T cell immunoglobulin and mucin-domain containing-3 (TIM-3), lymphocyte activation gene (LAG-3), programmed cell death protein-1 (PD-1) and cytotoxic T-lymphocyte-associated antigen 4 (CTLA-4) (**Figure 5**). Like the NCRs, these immunologic checkpoints have and share several ligands associated with cellular transformation and cancer. The ligands are discussed below, first alone, and then in the context of their receptors.

- Galectin-9 (Gal-9) is a C-type lectin that is bound by carbohydrate moieties found on membrane-bound proteins across several lymphocytes, including T cells and NK cells (291, 292). Gal-9 is expressed on the cell surface, and can be cleaved by metalloproteinases and secreted in soluble form (293). Gal-9 is involved in cell adhesion and migration (294, 295) and binds to TIM-3 on lymphocytes (296, 297).
- High mobility group box 1 (HMGB-1) is a nuclear protein that binds to and stabilizes DNA at steady state, but can translocate to the cytoplasm of stressed cells and act as a damage-associated molecular pattern (DAMP) that drives activation of innate immunity via toll-like receptors (298).
- Carcinoembryonic Antigen-Related Cell Adhesion Molecule 1 (CEACAM-1) is an immunoglobulin superfamily member most commonly found on epithelial cells, but also on immune cells with regulatory functions (299, 300). In cancer, expression of CEACAM-1 has been associated with both better and worse prognosis (301). In melanoma specifically, its expression is associated with aggressive metastasis (302) and immune exclusion (303).
- Phoshatidylserine is a component of the inner leaflet of healthy cell membranes, and contributes to maintenance of membrane integrity, cell signaling, adhesion and trafficking and apoptosis (304). When externalized – a consequence of apoptosis and cell death (305) – phosphatidylserine signals to phagocytose the damaged cell (304). Phosphatidylserine signaling generally promotes immune regulation, ostensibly to limit inflammation and prevent autoimmunity (306); in cancer, phosphatidylserine can interfere with lymphocyte-mediated tumor killing (307).
- HLA II presents exogenous antigens to helper (CD4^+^) T cells. HLA II is typically expressed on the surface of professional antigen presenting cells (308), and can be presented by cancer cells and antigen presenting cells in the tumor microenvironment (309).
- Lymph node sinusoidal endothelial cell C-type lectin (LSECtin) is an adhesion molecule that promotes tumor invasion and metastasis. LSECtin has a regulatory role, acting on cellular microRNAs to diminish immune cell activation (310).
- Programmed death ligand-1 and -2 (PD-L1/PD-L2) are type 1 transmembrane proteins of the immunoglobulin superfamily, commonly expressed on hematopoietic and non-hematopoietic cells such as endothelial cells, keratinocytes, and pancreatic islet cells (311). PD-L1 and PD-L2 expression is limited under homeostasis but upregulated in response to environmental stimuli, disease, and inflammatory cytokines including IFN-γ, TNF, and IL-6, likely to limit the extent of inflammation (312–314). PD-L1 overexpression has been observed in lung cancer, lymphoma, and pancreatic cancer (312, 315, 316) and associated with poorer outcomes, including worse overall survival and decreased progression free survival (317–319).
- CD80 and CD86 are members of the B7 receptor-ligand family and are expressed by antigen presenting cells and T regulatory cells (Tregs) (320, 321).

Reinvigoration of T cells has been the major goal of monoclonal antibodies against TIM-3, LAG-3 and PD-L1/L2, but NK cells may be also rescued by their inhibition in the cancer microenvironment. **TIM-3** signals for inhibition via five conserved tyrosine residues, and Bat-3 is associated with its transmembrane domain at steady state (322, 323). Known ligands for TIM-3 include Gal-9, HMGB-1, CEACAM-1, and phosphatidylserine. NK cells expressing TIM-3 have suppressed cell-mediated cytotoxicity and can be rescued with TIM-3 blockade (324, 325).

The specific role for **Gal-9**’s interactions with TIM-3 on NK cells is unclear, with reports of both stimulation for IFN-γ production by the NK-92 cell line (296) and immunoregulation in viral infections and pregnancy (326, 327), suggesting that its role may be nuanced or influenced by microenvironmental features. In T cells, Gal-9/TIM-3 binding triggers release of Bat-3, which liberates an immunosuppressive signal (328). TIM-3 signaling results in inhibition of T cell proliferation and cytokine productions, potentially leading to T cell death (291, 292).

In cancer, the impacts of TIM3:Gal-9 interactions on NK cells are similarly controversial, with reports alternately ascribing pro- and anti-tumor roles. TIM-3:Gal-9 interactions are associated with NK cell exhaustion and decreased cytotoxicity in AML and gastrointestinal tumors (329, 330). Increased Gal-9 expression is associated with worse overall survival, decreased progression-free survival, and increased metastasis in cancers of the liver (331), kidney (332) and virus-associated cancers (333). Conversely, and consistent with an activating role for TIM-3:Gal-9 interactions, higher expression of Gal-9 is associated with less tumor dedifferentiation and metastasis in cervical cancer (334). Gal-9 drives apoptosis of melanoma, leukemia, and lymphoma cell lines (335, 336). Higher Gal-9 expression is associated with superior outcomes in patients with gastric cancer (337) and triple-negative breast cancer (338).

**LAG-3** is a member of the immunoglobulin superfamily receptors, and binds MHC class II, and LSECtin (339–343). On T cells, LAG-3 is induced by cell activation, ostensibly to enable control of ongoing lymphocyte responses (344, 345), associates with the TCR and binds HLA II with higher affinity than CD4, to regulate signaling and inhibit proliferation (344–346). LAG-3 is known to be expressed by NK cells (347) with the highest expression being reported on activated, adaptive, and mature NK cells (348).

The expression of MHC II in the tumor microenvironment is typically associated with improved immune cell infiltration and patient outcomes (349, 350). Expression of LAG-3, on the other hand, is well established as a marker of increased tumor progression and aggressiveness across cancer types (351–353). As a result, therapies directly targeting LAG-3 are currently being explored, both as monotherapy and in combination with immune checkpoint blockade. While studies evaluating LAG-3 therapies rarely profile NK cells, one study evaluating the response of patients with melanoma to a combination of anti-LAG-3 and anti-PD-1 found that adaptive LAG-3^+^ NK cells were most prominent in those who responded to immunotherapy (348). This data supports the role for NK cells influencing response to checkpoint inhibitors. Interestingly, this has also been suggested by earlier work where NK cells isolated from a murine model with LAG-3 deficiency exhibited defects in NK cell mediated anti-tumor immunity (354). Additional research is needed to better understand the role and importance of LAG-3 in NK cell cancer killing.

**PD-1** is expressed most prominently on T cells, and has recently been described to be present, but at lower densities on mouse and human NK cells (355, 356). Interference with PD-1/PD-L1/2 signaling with monoclonal antibodies on these mouse NK cells increases their target cell cytotoxicity, confirming a parallel function to that described for T cells (357, 358). PD-1 expression on human NK cells is not yet well understood and seems to be limited to the adaptive NK cell subset (359–361). Nonetheless, blockade of the PD-1/PD-L1 pathway improves NK cell responsiveness both *in vitro* and *in vivo* (362, 363). Blocking PD-L1^+^ NK cells with anti-PD-L1 improves degranulation and cytokine production, as well as control tumor *in vivo*, indicating that NK cells may contribute to the success of checkpoint inhibition independent of PD-1 expression (364).

Monoclonal antibodies that interfere with PD-L1/L2-PD-1 interactions have become part of treatment for an array of tumors. Durvalumab and atezolizumab (both anti-PD-L1 monoclonal antibodies) are approved as first-line treatments for non-small cell lung cancer (365). Further, durvalumab is approved for bladder cancer and atezolizumab is approved for treatment of triple-negative breast cancer and liver cancer (366–369). Pembrolizumab (anti-PD-1) is also approved for use in MSI-H/dMMR cancers (319). Whether and to what extent these impact NK cell function with impacts on cancer control in patients is unknown.

**CTLA-4** is a member of the B7/CD28 family and is constitutively expressed on regulatory T cells, as well as upregulated on other T cell subsets upon activation (370). CTLA-4 inhibits CD28 signalling (371), and T cell activation, through the competitive binding of its ligands **CD80/CD86** (372). Expression of CTLA-4 has been reported in both activated mouse and human NK cells (355, 373, 374). In healthy human donors, CTLA-4 expression on NK cell populations is low, and associated with decreased production of activating cytokines and an increase in IL-10 production (355). CTLA-4 expression was found on mediastinal lymph node-derived NK cells of NSCLC patients (375) and on tumor infiltrating NK cells in early-stage lung cancer (376).

The exact role of CTLA-4 expressing NK cells has not yet been fully elucidated. Nonetheless, NK cells have been shown to play a role in the success of anti-CTLA-4 therapies. In melanoma, patients’ response to ipilimumab correlated with an activated NK cell signature (377). Further, ipilimumab was shown to directly bind NK cells through immunofluorescence microscopy. Along with directly binding ipilimumab, NK cells further target CTLA-4 expressing Tregs *in vivo* and target them through ADCC (378, 379).

Monoclonal antibodies against CTLA-4 are currently in the clinic both alone and in combination with other checkpoint inhibitors. Ipilimumab was first approved as a single-therapy treatment in melanoma (380) and has since been approved as a combination treatment with nivolumab in several cancer types, including colorectal (381), liver (382), renal cell (381), and lung cancer (383). Tremelimumab has recently been approved in combination with durvalumab for the treatment of patients with unresectable liver cancer (384).

Altogether, the immunologic checkpoints that impact T cells may also inhibit NK cells (and be rescued by immune checkpoint blockade). Whether these classical checkpoints are effective targets, or other mechanisms of NK cell inhibition would enable superior anticancer activity remains to be defined.

## 3 Leveraging the multifaceted features of NK cells for immunotherapy

Though most studies consider the role of NK cell receptor-ligand partnerships in isolation, individual NK cells express constellations of receptors, and their potential impacts should be considered as a composite response. For instance, even strong signals for activation can be outweighed by concurrent signals for inhibition via KIR-HLA interactions (33). Moreover, NK cell function, receptor expression and arming are impacted by local signals, and can change as the environment does (8, 385). Finally, strategies to intervene and manipulate NK cells must also consider the impacts on other cells; for instance, delivery of receptor agonists and cytokines may have impacts beyond their direct impacts on NK cells (386, 387).

Although immunotherapies have dramatically changed outcomes for people with cancer, there remains significant mortality from the disease, refractory cancers, and relapse of cancers with acquired therapeutic resistance (388–390). The tumor microenvironment is often highly immunosuppressive, and tumor cells themselves diversified, so effective treatment with a single-targeting agent is challenging. The variety of functional capabilities and mechanisms of effector:tumor engagement that direct NK cell function make them intriguing targets for immunotherapy, because they may provide a mechanism for agile and ongoing recognition of plastic cancer cells. Already, NK cells are recruited by standard of care treatments and immunotherapeutic strategies, including those that use monoclonal antibodies against tumor antigens and immunologic checkpoint blockade. Strategies exist to expand, engineer, and transfer allogeneic NK cells. Hence, it is feasible to recruit and use NK cells as immunotherapy; the next challenge will be to adequately direct them for cancer killing. We expect this will be achieved by targeting multiple NK cell features simultaneously.

Standard approaches to treatment may be combined with NK cell-targeting therapies to achieve more complete tumor control. Fas agonism, for example, is rendered less-toxic in the context of chemotherapy, so combining these agents may create a synergistic impact to allow greater tumor recognition and control (243). Recognizing that one of NK cells’ intrinsic functions is to respond to “stress” ligands, it is unsurprising that inflammation-inducing therapies can support NK cell activity. For example, radiation induces CXCL8 production in pancreatic tumors, attracting CD56^dim^ NK cells; this associates with prolonged survival (35). Other studies have reported NK cell mediated responses to oncolytic virus infected cells (391), and chemotherapy (34). These treatments all induce inflammatory responses, which may serve to alter the immunosuppressive tumor microenvironment and attract and activate NK cells.

Creating a supportive environment for NK cell function may be facilitated by provision of **immune modulating cytokines**. Early cytokine therapies included systemic delivery of IL-2 (392), but the concentration of IL-2 needed to achieve meaningful clinical responses was associated with severe adverse toxicities (393). In addition to T cells, NK cells can be activated by IL-2, but high-dose IL-2 can ultimately deprive NK cells of IL-2 as it instead supports regulatory T cell activation and expansion (394). These initial findings led to the attempted use of lower dosing, less potent analogues, *ex vivo* cytokine treatment of NK cells, or more specific cytokines signals, including IL-15, which strongly supports NK cell proliferation and activation.

IL-15 is most potent in the context of its receptor, IL-15Rα. As therapy, IL-15 and its receptors have been engineered to enhance stability and efficacy. These constructs include the IL-15 super-agonist complex (ALT-803), which is a complex containing a mutated IL-15 (N7D) and IL-15Rα (395). ALT-803 increases NK cell proliferation in the ascites of ovarian cancer patients and increases healthy donor NK cell function against ovarian cancer cell lines (395). When combined with IL-12 and IL-18, IL-15 can induce the adaptive, memory-like NK cell features, including enhanced anti-cancer function (396). More recently, a heteromeric fusion protein complex (HFPC) platform combined IL-12, IL-15, and IL-18, that enhanced primary NK cell proliferation more efficiently compared to the cytokines administered (397).

Immune stimulating agents, including agonists of the stimulator of interferon genes (STING) pathway can likewise achieve an inflammatory and immune-permissive microenvironment (398). STING silencing is a mechanism used by tumors to quiet immune responses (399, 400), and activating STING can enhance NK-cell mediated immunotherapy (401, 402) and NK cell trafficking via CXCR3 (401, 402). IL-2 and STING-agonists together support T and NK cell activation against treatment-refractory mouse tumor models (401, 403). Potentially identifying a mechanism that contributes to these responses, a recent single-cell approach identified that STING mediates its anti-tumor immune stimulating impact, in part, through CXCR3 upregulation and the subsequential recruitment and activation of NK cells (402).

Beyond general signals for inflammation, NK cells can contribute to antigen-specific anti-cancer responses via ADCC driven by therapeutic **monoclonal antibodies**. More recently, these have been created with cytokines and other agents to drive activation and killing by NK cells simultaneously. One such **fusion protein**, for example, combines the ALT-803 backbone with rituximab (404). In mouse models, this compound, “N-803” induces NK cells for increased secretion of cytokines, chemokines and growth factors, cytotoxicity, and control of rituximab resistant bone lesions when compared to those cultured or treated with rituximab and ALT-803 while NK cells exhibited enhanced expression of NKG2D, CD16, NCRs, and enhanced cytotoxicity (405). Other fusion proteins focus on enhancing signaling through activating receptors on NK cells. For example, CD123-NKCE, is a TriKE recently developed to bind CD123 on acute myeloid leukemia, while simultaneously signalling through NKp46 and CD16a on NK cells (232). This engager prevented CD64-mediated ADCC inhibition which is a usual mechanism of evasion undertaken by acute myeloid leukemia cells. These strategies direct NK cell mediated ADCC while also focusing on enhancing the NK itself.

NK cells express the immune checkpoints, PD-1 and LAG-3, and **immune checkpoint blockade** may enable NK cell anti-tumor responses. In the blood of patients with metastatic melanoma treated with relatlimab (anti-LAG-3) and nivolumab (anti-PD-1) the “adaptive” NK cell subset exhibited the highest LAG-3 expression, and responding patient NK cells were activated with treatment (348). A pre-clinical approach leverages the extensive availability of PD-L1 in the tumor microenvironment and employs NK cells equipped with a chimeric switch receptor linking PD-1 to activating domains CD3ζ, DAP10, or DAP12 (406). These cells had superior cytotoxicity towards PD-L1^+^ target cells compared to wildtype NK cells (406). In pre-clinical studies, inhibition of other immune checkpoints TGFβ and CIS (an NK cell IL-15 signalling checkpoint encoded by *CISH*) simultaneously results in enhanced NK cell activation and decreased MC38 colorectal cancer tumor burden in mice (407). Similarly, *CISH* is another member of the suppressor of cytokine signaling (SOCS) family encoded by *CISH* that when targeted, improved NK cell effector functions (408).

A major advantage of NK cells is that they can be adoptively transferred across allogeneic barriers and expanded extensively *ex vivo*, opening the possibility of “off the shelf” cancer therapy. Strategies to select and/or engineer NK cells with the greatest anti-cancer potential are still in development and approaches that maximize NK cell activation against tumors will likely be the most effective against cancer. For example, recent work demonstrating that isolating single-KIR^+^NKG2C^+^ NK cells from donors harboring large adaptive NK cell subsets could be used to optimize response against HLA-mismatched acute myeloid leukemia (409). Other cellular sources for NK cells, including umbilical cord blood stem cells or induced pluripotent stem cells (iPSCs) may provide further flexibility and opportunities to tailor NK cells as adoptive cell therapy [reviewed recently by (410, 411)]. For example, NK cell metabolism could be reprogramed in iPSC derived NK cells through the deletion of CISH, which normally opposes IL-15 signaling, to enhance *in vivo* persistence and efficacy (412).

NK cell expansion protocols provide an ideal platform for modulating NK cell populations through pharmacologic interventions such as **small molecule inhibitors**. There are several small molecule inhibitors available that target proteins used by NK cells to regulate cell signalling. For example, glycogen synthetase kinase (GSK)3 inhibitors can halt GSK3 mediated NFκB inhibition, thereby promoting NK cell activation (413). Indeed, administering a GSK3 inhibitor, CHIR99021, to IL-15 expanded adaptive NK cells enhanced cytokine production, natural cytotoxicity, and antibody-dependent cytotoxicity (414). These small molecule inhibitors can also be harnessed to enhance NK cell resistance against tumor-mediated suppression. Canonical TGF-β signaling suppresses NK cell function and remains a barrier to intra-tumoral NK cell activation (415). TGF-β signaling is facilitated through SMAD3, which can be inhibited through small molecule inhibitor SIS3 (416). Indeed, the use of SIS3 *in vitro* and *in vivo* has demonstrated the ability to release E4BP4/NFIL3 NK cell differentiation and promote NK cell mediated lung tumor control (417).

**Engineered chimeric antigen receptor (CAR)-NK** cells allow durable, antigen-directed targets alongside the anticancer activity of NK cells and offer the combined advantages of NK cells and potent antigen targeting through CAR. CAR-NK have exhibited exceptional efficacy. In a clinical trial employing CD19-targeting CAR-NK cells in patients with lymphoid malignancies, CAR-NK cells were well-tolerated, and generated complete remission in 7/11 treated patients (38). Combined strategies to maximize key features of NK cells are in their infancy, but possible and may further enhance the efficacy of NK cellular therapy. For example, CAR-NK cells have been further modified to enhance ADCC can by inclusion of non-cleavable CD16 and a membrane-bound IL-15 fusion molecule (358). There are many combinations that are feasible, but following the biology of NK cells and the tumors against which they act may help to design rational, bespoke approaches to comprehensive tumor targeting.

## 4 Concluding remarks

NK cells are equipped with a toolkit of germline-encoded activating and inhibitory receptors, which act together to integrate incoming signals. Since the receptors on the NK cells that comprise a person’s repertoire are variable, they provide extensive diversity to recognize a variety of target cell phenotypes. NK cells are critical agents of immunosurveillance and participate in existing approaches to treat cancer.

The next generation of immunotherapies are multivalent: they simultaneously target more than one feature of tumors to prevent immune escape. Strategies to quantify the strength of signaling associations based on both NK cell receptors and the ligands present will be required to prioritize targets. Understood, these will enable development of multitargeted, precision NK cell-based cancer immunotherapies.

## Author contributions

SN: Conceptualization, Validation, Visualization, Writing – original draft, Writing – review & editing. EC: Conceptualization, Writing – original draft, Writing – review & editing. SL: Conceptualization, Writing – original draft, Writing – review & editing. LW: Writing – original draft, Writing – review & editing. JB: Conceptualization, Funding acquisition, Resources, Supervision, Validation, Visualization, Writing – original draft, Writing – review & editing.
